# Editorial: Metabolic Regulation of Cardiac and Vascular Cell Function: Physiological and Pathophysiological Implications

**DOI:** 10.3389/fphys.2022.849869

**Published:** 2022-02-15

**Authors:** Mohsin Khan, Mirko Völkers, Adam R. Wende

**Affiliations:** ^1^Center for Metabolic Disease Research, Lewis Katz School of Medicine, Temple University, Philadelphia, PA, United States; ^2^Department of Cardiology, Angiology and Pneumology, University Hospital Heidelberg, Heidelberg, Germany; ^3^DZHK (German Centre for Cardiovascular Research), Partner Site Heidelberg-Mannheim, Heidelberg, Germany; ^4^Department of Pathology, University of Alabama at Birmingham, Birmingham, AL, United States

**Keywords:** cardiac cell, metabolism, vascular cells, heart, hypoxia

This Research Topic gathers a collection of five original and review articles that provide recent summaries and novel information regarding the role of metabolism in regulating cardiac and vascular cell function. The mammalian heart is a pump that requires a constant supply of energy to maintain function. Cardiac metabolism plays a pivotal role in driving molecular and cellular changes in the heart in response to physiological and pathological stresses. The adult heart generates >95% of its ATP primarily by oxidative phosphorylation in the mitochondria while the remaining 5% comes from glycolysis, or to a lesser extent from the citric acid cycle. In contrast, during development cardiac tissue relies on glycolysis to meet its energy demands during development. However, the postnatal heart undergoes a rapid shift in metabolism to oxidative phosphorylation in response to changes in oxygen tension and maturation. Injury to the adult heart is accompanied by a shift from fatty acid oxidation to glucose use, like a fetal metabolic state, promotes oxygen efficiency for ATP synthesis, considered to be beneficial particularly under ischemic cardiac injury where oxygen supply is limited. Recently, metabolism has been linked to the regulation of gene expression in cardiac cells with levels of several metabolites altered in response to physiological and pathological stresses in the heart.

## Endothelial Dysfunction by Dysferlin and the Effect on Atherosclerosis

Atherosclerosis is the primary cause for ischemic heart disease and stroke (Lusis, 2000) meriting the need to define causal mechanisms for development of atherosclerosis (Ziaeian and Fonarow, 2016). In this context, the role of Dysferlin on atherosclerosis and plasma lipoprotein composition was investigated (White et al.). Dysferlin is a plasma membrane protein, regulating vesicle trafficking, and repair of the plasma membrane in muscle tissue (Bulankina and Thoms, 2020). Dysferlin is also expressed in endothelial cells (Sharma et al., 2010), but the role of dysferlin during atherosclerosis was unknown. The study by White et al. now establishes that dysferlin is highly expressed in atheromatous medial and intimal cells, but loss of dysferlin did not affect hyperlipidemia mediated atherosclerosis in the ApoE model. The authors used a global dysferlin knockout (KO) mouse model with or without expression of ApoE and fed the mice with a high fat diet. Interestingly, dysferlin KO mice showed differential lipoprotein composition compared to control mice, but atherosclerotic plaques were similar compared to wild-type control mice. Though several mechanistic questions remain, such as the cell type specific role of dysferlin in atherosclerosis, which could be addressed in cell type specific knockout mouse models, the study presented here provides the first evidence that patients with dysferlinopathies, caused by mutations to dysferlin, might be dyslipidemic but not at greater risks for atherosclerosis.

## Endothelial Dysfunction and SARS-CoV-2

Two-years since the discovery of COVID19, the pandemic is not over. With over 271 million cases and more than 5.3 million deaths worldwide, SARS-CoV-2 has resulted in a global health crisis. Clearly, several pathomechanisms are involved in COVID19, and among them lung endothelial dysfunction is a key player in the disease evidenced by the fact that many complications associated with COVID19 such as thrombosis, and acute respiratory distress syndrome are directly related to endothelial dysfunction. In a comprehensive review article, Chang et al. summarize the role of endothelial dysfunction and the interplay to chronic oxidative stress.

## Cardiovascular Disease and Endothelial Metabolism Regulation by Hypoxia

Cardiac tissue during postnatal development is hypoxic which drives glycolytic metabolism. Postnatal development is accompanied by changes in oxygen tension in response to ambient oxygen altering metabolic signaling in cardiac cells. Moreover, regions of low oxygen tension within cardiac tissue in the adult heart support cardiac stem cells and cardiomyocytes with regenerative properties. Several signaling pathways have been identified in response to changes in oxygen tension in the heart and their role in regulating molecular and cellular function in cardiac cells. In this respect, Ullah and Wu review the literature regarding the role of a master regulator of hypoxia responses, i.e., hypoxia-inducible factors (HIFs) in endothelial cells focused on metabolism and cardiovascular disease outcomes.

## Cardiac Metabolic Complications Associated With Aging

Aging is a significant risk factor for cardiovascular disease progression. Interestingly, changes in cardiac metabolism are tightly linked to development and the aging process. The mini review by Sithara and Drosatos summarizes some of the mechanisms that contribute to cardiovascular complications associated with aging. Authors discuss cardiac metabolism both in healthy heart and in aging heart as well as how its decline is impacted by mitochondrial dysfunction and systemic changes. Authors highlight cardiac ketone body oxidation, autophagy (and the related mitophagy), and oxidative signaling associated with mitochondrial function and how inflammation can exacerbate development of age associated cardiomyopathy. Importantly, they highlight that despite decades of research on cardiac metabolism, additional research as it relates to the progression of cardiac aging is required especially as it relates to cardiovascular complications in the aging human population.

## Cardiac Metabolic Complications Associated With Cancer

Cancer is increasingly linked to cardiovascular disease progression. Although several early studies found cardiotoxic effects of chemotherapies, more recently it is clear that other mechanisms are impacting cardiovascular health. The mini review by Finke et al. summarizes several recent findings in rodent models and clinical studies that reveal mechanisms for crosstalk between cancer cells and the heart. Authors focus on changes associated with metabolic switches described in the prior mini review associated with aging, i.e., fatty acid to glucose utilization. More specifically, authors link critical transcriptional co-regulators of mitochondrial function and metabolism, onco-metabolites, and both inflammatory and non-inflammatory cytokines to disease progression. Several key studies defining epigenetic mechanisms that may be involved in both disease states, their crosstalk, in the form of non-coding microRNAs are discussed. Authors further point out which of these pathways have been explored specifically in the context of cardiovascular disease, in cancer, or at their intersection. Taken together this summary of the current literature highlights the clinical importance of understanding the molecular underpinnings in treating cancer patients with or without underlying cardiovascular disease as there may be synergistic effects that require personalized treatment options.

## Author Contributions

All authors listed have made a substantial, direct, and intellectual contribution to the work and approved it for publication.

## Funding

This work was supported by National Institute of Health grant HL135177, American heart Association Transformational Project Award (20TPA35490355 to MK) and National Institute of Health grants (HLHL133011 to AW), and (DFG VO1659-2/1 to MV).

## Conflict of Interest

The authors declare that the research was conducted in the absence of any commercial or financial relationships that could be construed as a potential conflict of interest.

## Publisher's Note

All claims expressed in this article are solely those of the authors and do not necessarily represent those of their affiliated organizations, or those of the publisher, the editors and the reviewers. Any product that may be evaluated in this article, or claim that may be made by its manufacturer, is not guaranteed or endorsed by the publisher.

## References

[B1] Bulankina A. V. Thoms S. (2020). Functions of vertebrate ferlins. Cells 9, 534. 10.3390/cells9030534 32106631 PMC7140416

[B2] Lusis A. J. (2000). Insight review articles. Nature 407, 233–241. 10.1038/35025203 11001066 PMC2826222

[B3] Sharma A. Yu C. Leung C. Trane A. Lau M. Utokaparch S.. (2010). A new role for the muscle repair protein dysferlin in endothelial cell adhesion and angiogenesis. Arterioscler. Thromb. Vasc. Biol. 30, 2196–2204. 10.1161/ATVBAHA.110.208108 20724702 PMC2996267

[B4] Ziaeian B. Fonarow G. C. (2016). Epidemiology and aetiology of heart failure. Nat. Rev. Cardiol. 13:368–78. 10.1038/nrcardio.2016.25 26935038 PMC4868779

